# Collagens and Cancer associated fibroblasts in the reactive stroma and its relation to Cancer biology

**DOI:** 10.1186/s13046-019-1110-6

**Published:** 2019-03-06

**Authors:** Neel I. Nissen, Morten Karsdal, Nicholas Willumsen

**Affiliations:** 1 Biomarkers and Research, Nordic Bioscience A/S, Herlev Hovedgade 205-207, 2730 Herlev, Denmark; 2 Biotech Research & Innovation Centre (BRIC), University of Copenhagen, Ole Maaløes vej 5, 2200 Copenhagen N, Denmark

**Keywords:** Oncology, Extracellular matrix (ECM), Collagens, Cancer-associated fibroblasts (CAFs), Desmoplasia, Biomarkers, Liquid biopsy

## Abstract

The extracellular matrix (ECM) plays an important role in cancer progression. It can be divided into the basement membrane (BM) that supports epithelial/endothelial cell behavior and the interstitial matrix (IM) that supports the underlying stromal compartment. The major components of the ECM are the collagens. While breaching of the BM and turnover of e.g. type IV collagen, is a well described part of tumorigenesis, less is known regarding the impact on tumorigenesis from the collagens residing in the stroma. Here we give an introduction and overview to the link between tumorigenesis and stromal collagens, with focus on the fibrillar collagens type I, II, III, V, XI, XXIV and XXVII as well as type VI collagen. Moreover, we discuss the impact of the cells responsible for this altered stromal collagen remodeling, the cancer associated fibroblasts (CAFs), and how these cells are key players in orchestrating the tumor microenvironment composition and tissue microarchitecture, hence also driving tumorigenesis and affecting response to treatment. Lastly, we discuss how specific collagen-derived biomarkers reflecting the turnover of stromal collagens and CAF activity may be used as tools to non-invasively interrogate stromal reactivity in the tumor microenvironment and predict response to treatment.

## Introduction

The ECM is an extensive part of the microenvironment in all tissues. It consists of a non-cellular meshwork of proteins, glycoproteins, proteoglycans and polysaccharides. When structured in an orderly manner, the ECM provides a physical scaffold for its surrounding cells, bind growth factors and regulate cell behavior.

The ECM can be divided into two matrices: the basement membrane (BM) and the interstitial matrix (IM). Under healthy conditions, the BM is a well-structured membrane underlining epithelial and endothelial cells and separating them from the IM. When fully assembled the BM provides structural support to underlining cells and regulate cell behavior. The IM makes up the main stroma and plays a major role in cell migration, cell adhesion, angiogenesis, tissue development and repair [1].

**Fig. 1:**
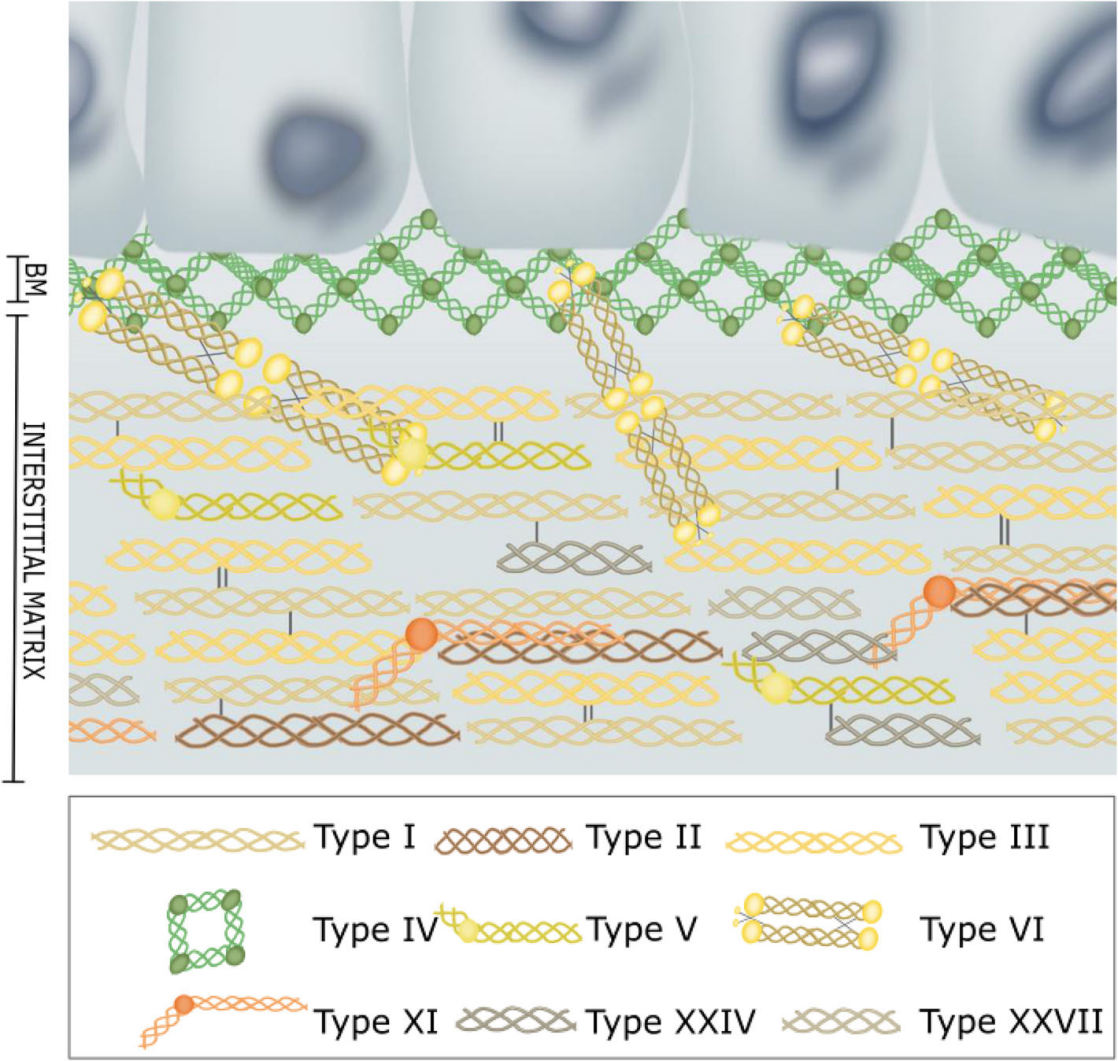
Collagens within the basement membrane and interstitial matrix. Schematic drawing of the structure and localization of network-forming collagens (type IV collagen), beaded filament (type VI collagen) and fibril-forming collagens (type I, II, III, V, XI, XXIV and XXVII collagens)

The major proteins in the ECM are collagens, which constitutes up to 30% of the total protein mass in the human body [2]. The collagens are organized in a relaxed meshwork surrounded by proteins such as elastin and glycoproteins causing a resilience to extensive tensile strength [2]. Of today, 28 different collagens have been identified creating a unique ECM composition in different tissues. The 28 collagens can be divided into several distinct subgroups, where the so-called fibrillar-forming collagens and the network-forming collagens have been most extensively characterized [3]. The major components of the BM are the network-forming collagens such as type IV and type VIII collagen whereas the IM is dominated by the fibrillar-forming collagens type I, II, III, V, XI, XXIV, XXVII and the beaded filament type VI collagen synthesized by the fibroblasts receding in the stroma [4–8]. These collagens are not just collagens but individual structures creating a complex network that interact with each other and the surroundings (Fig. 1).

In the healthy tissue there is an ongoing ECM remodeling to maintain tissue integrity and function, e.g. new collagens synthesized that replaces older proteins that are degraded. The collagen production and assembly in healthy tissue is highly regulated by a perfect counterbalance of metalloproteinases (MMPs) and inhibitors of MMPs as well as a controlled activity of other enzymes such as lysyl oxidases (LOX) [9, 10].

During cancer, the ECM-dynamics are skewed. It is well established that cancer cells secrete high amounts of MMPs, which in turn remodel and degrade the BM. The remodeling of the BM leads to a complex chaos of pro- and antitumor signals from degradation products. The role of type IV collagen turnover, within the BM, has been extensively studied in relation to tumor biology. Several studies have shown that proteolytic cleavage of collagen IV can expose so-called cryptic domains, which are normally hidden when collagen IV is fully assembled [11–14]. Similar things have been seen with other BM collagens e.g. type XVIII collagen [15]. Depending on the context, these cryptic sites have both pro- and anti-tumor effects; still the turnover and degradation of BM collagens are intrinsically associated with the invasive phenotype of malignant cells [11].

**Fig. 2:**
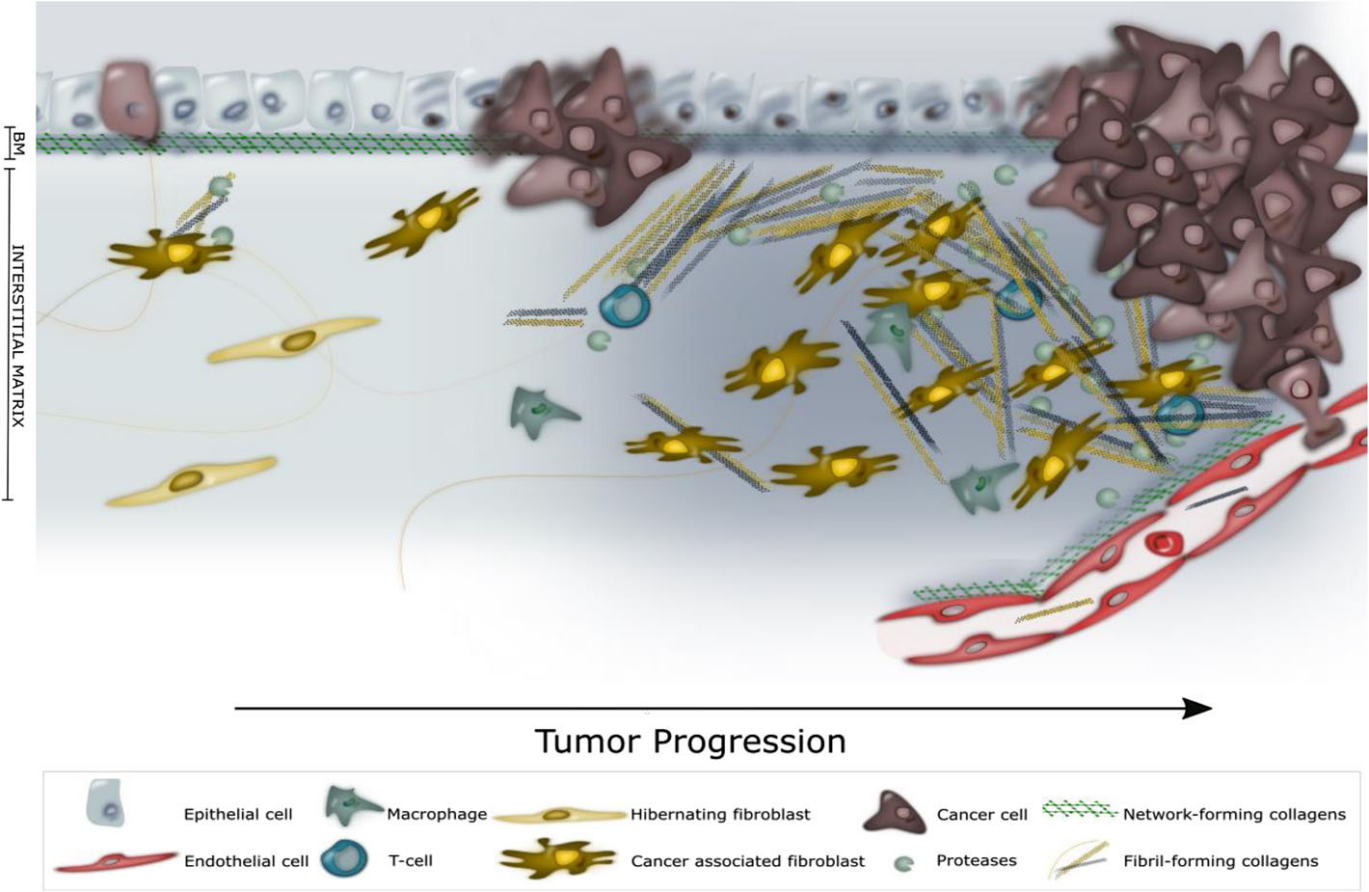
The extracellular matrix during tumor progression. As the cancer cells invade the basement membrane (BM) the interstitial matrix (IM) becomes more and more desmoplastic characterized by an increased activity of cancer-associated fibroblasts (CAFs) and augmented volume of cross-linked type I, II, III, V, VI, XI, XXIV and XXVII collagens. In the later stages of tumor progression, desmoplasia pre-dominate the tumor microenvironment with signals from CAFs and IM collagens stimulating and sustaining the tumor progression

Tumor cell invasion through the BM expose malignant cells to the IM and the fibroblast derived collagens; type I, II, III, V, VI, XI, XXIV and XXVII collagens. Type I, II, III, V, XI, XXIV and XXVII collagens are all fibrillary collagens embedded in the IM, whereas type VI collagen is found in the interface between the BM and the IM. Emerging evidence indicate a high impact of fibroblast-derived collagens and so-called cancer associated fibroblasts (CAFs) in tumorigenesis [16, 17]. During tumor progression, CAFs are the major players in the dysregulated collagen turnover leading to tumor fibrosis (desmoplasia) characterized by excessive collagen depositions in the surroundings of the tumor [18, 19]. The collagens are often crosslinked and linearized leading to increased stiffening of the tissue (Fig. 2). This elicits behavioral effects on surrounding tumor cells, and regulate cell proliferation, differentiation, gene expression, migration, invasion, metastasis and survival and hereby the collagens are directly affecting the hallmarks of cancer [20]. In support, tumor tissue, containing a large amount of these fibroblast derived stromal collagens is directly correlated with poorer outcome for the patient [21–25].

Here we give an introduction and overview to the link between tumorigenesis, fibroblasts derived collagens and CAFs.

### CANCER ASSOCIATED FIBROBLASTS – Key players in cancer progression and desmoplasia

New insight into the role of CAFs have shown that these cells play a key-role in cancer progression. In the tumor microenvironment, transforming growth factor beta (TGF-β), platelet-derived growth factor and fibroblast growth factor-2, among others, secreted from malignant cells, attracts fibroblasts from neighboring tissue as well as aid in the transformation of normal fibroblast to CAFs within the tumor tissue [26–30]. Up to 80% of the normal fibroblasts in breast tissue acquire the CAF phenotype during cancer progression [17]. Interestingly, CAFs can also originate from epithelial cells [31], immune cells and endothelial cells [32] emphasizing the complexity of this cell type. The CAF phenotype is characterized by changes in morphology and increased expression of myofibroblast markers such as alpha-smooth muscle actin (α-SMA), Vimentin, type XI collagen, fibronectin, fibroblast specific protein 1 (FSP-1) and fibroblast activating protein (FAP) [33]. Furthermore, CAFs show increased production of IM collagens [34, 35]. It is an ongoing discussion whether distinctive features between CAFs and myofibroblasts exist. The literature focusing on this topic is scarce and it seems that there is no consensus on what define quiescent fibroblasts, myofibroblasts and CAFs. Myofibroblasts (hepatic myofibroblasts) and CAFs (fibroblasts isolated from liver cancer patients) expressing α-SMA and Tenascin-C show similar apoptosis signaling compared to fibroblasts not expressing α-SMA and Tenascin-C (defined by the authors as quiescent fibroblasts) [29]. However, in another study performed by Öhlund and colleagues, it was shown that the transcriptional profiles between myofibroblasts (pancreatic stellate cells grown in monolayer) and CAFs (pancreatic stellate cells cocultured with tumor organoids) differs [36]. Thus, the difficulties in defining what a CAF is and compare these cells/cell states [37] to other cells is still an ongoing battle. To further complicate things, many studies have shown that different CAF subtypes exist based on differences in protein expression, paracrine signaling, tumorigenicity, invasion profile, ECM modifying capacities etc. [16, 19, 27, 33, 35, 36, 38–40].

Several studies indicate that CAFs modulate epithelial transformation and promote cancer progression. As one example, CAFs have shown to initiate malignant transformation in non-malignant cells through overexpression of estrogen, TGF-β and hepatocyte growth factor [41, 42]. As another example, a more mesenchymal phenotype has been observed for non-malignant prostate cells, when co-cultured with CAFs [43]. In addition to these in vitro examples, the initiation of cancer, by CAFs, have also been shown in vivo, where the injection of non-tumorigenic prostate cells, co-cultured with CAFs, lead to the formation of large tumors. On the contrary, no formation was evident when cells were cultured with normal fibroblasts [41]. Other than initiating cancer, CAFs have also been shown to sustain cancer progression and induce angiogenesis. Breast tumor tissue, isolated from mice, containing abundant amounts of CAFs shows increased vascularity compared to tissue with normal fibroblast [44]. Glentis and colleagues, suggest that CAFs play a role in the invasion of cancer cells through the BM by pulling and stretching the BM resulting in small wholes which the cancer cells can squeeze through [39]. They also showed that especially invasive tumors from colon cancer patients are surrounded by a thick capsule of CAFs, which further suggests CAF involvement in invasion [39]. In line with this, CAFs also play a role in metastasis. Lung cancer cells treated with media from CAFs have increased migration potential compared to cells treated with media from normal fibroblasts [45]. This is further supported, by a study showing that cervical cancer cells co-transplanted with CAFs into mice leads to lymph node metastasis. In contrast, injections without CAFs do not lead to lymph node metastasis [46]. The association between CAFs and lymph node metastasis has also been shown in esophageal squamous cell carcinoma in humans [47]. Several studies have also shown that CAFs play a role in inflammation by modulation of inflammatory components which promote tumor growth and metastasis [36, 48–50] (reviewed by [51]). Thus, these examples show the important role CAFs play in initiating and sustaining epithelial transformation and cancer progression across many different solid tumor types.

Aside from directly affecting cancer cells, CAFs are also major contributors to desmoplasia and remodeling of the ECM. Recent evidence indicates that CAFs modulate the desmoplastic reaction by affecting a wide variety of ECM proteins during tumorigenesis. A study has shown that CAFs take part in the assembly of fibronectin, which is highly abundant in the ECM and strongly involved in metastasis [52]. CAFs also express high amounts of the major ECM component hyaluronic acid, which has shown to encompass many structural and biological functions in tumor progression [53]. The oncogenes YAP/TAZ are suggested as being part of the remodeling processes exerted by CAFs. When the ECM becomes stiff, YAP/TAZ gets transcriptionally active and promote CAF function which further stiffens the ECM (reviewed in [54]) [55]. The regulation of YAP/TAZ, resulting in CAF activity, is further regulated by the so called Rho family of small GTPases, which plays a role in CAF functioning and myofibroblast signaling [54, 56, 57].

Some of the major steps in desmoplasia are cross-linking of collagens, fiber elongation and fiber realignment, which are associated with poor survival in cancer patients [35, 58]. CAFs secrete increased amounts of MMPs and LOX-proteins, which catalyze these steps [19, 35]. CAF secreted MMPs also play a key role in neovascularization because of the release of VEGF from degraded matrix [11, 39, 59]. ECM proteins secreted and modulated by CAFs further recruit other cell types such as immune cells, which promote tumor progression [26, 27]. Finally, a key step in desmoplasia, is the increased expression of fibroblast-derived collagens within the stroma. The accumulation of collagens, accompanied by increased cross-linking and stiffening of the tissue increase interstitial fluid pressure [60]**.** This effect has been shown to reduce drug delivery of chemotherapy and immunotherapy [60]. The stiffened tissue also play a role in tumor cell invasion, as the cross-linked collagens can create paths for the tumor cells to travel on [61].

Although consensus is that desmoplasia is a pro-tumorigenic event, results have emerged from mouse studies that have raised debate in the field. In one study, it has been shown that when the stromal content was reduced by deleting the sonic hedgehog protein in a pancreas cancer mouse model, the mice had more aggressive tumors as compared to control mice [62]. This was supported by similar findings, showing that the depletion of CAFs in mice led to much more aggressive tumors [63]. These findings do not exclude that desmoplasia is pro-tumorigenic, but suggest that a homeostatic restoration of the desmoplastic stroma, rather than its ablation, may be the best approach for eliminating tumor progression, as also suggested by Froeling and Kocher [64]. To further complicate matters, it has been suggested that some CAF subsets promote cancer, while others inhibit cancer [16, 65]. Albeit CAF biology and desmoplasia is complex, tumor tissue containing high amounts of CAFs have been reported to correlate with poor patient outcome in many different cancer types including colorectal, breast, tongue and esophageal cancer [66–70].

### Fibroblast derived stromal collagens and their contribution to tumorigenesis

While extensive research is currently going in the direction of CAF phenotype and their prognostic aspects, less in known regarding the collagens they produce. Are there functional differences in the collagen profile of tumors and does ‘good’ and ‘bad’ collagens exist in the tumor microenvironment as has been described for fibrosis [71], i.e. are collagen components originating from CAFs affecting tumor progression?

Collagens, and especially fibroblast-derived collagens (fibrillar collagens and the beaded filament type VI collagen), are extremely important in cancer. Most of these collagens are upregulated in cancer on both gene and protein level. They all modulate crucial steps in tumorigenesis such as proliferation, apoptosis, angiogenesis, invasion and metastasis. For many of the fibroblast-derived collagens specific chains of the collagens and pro-collagens have shown to be the effectors. Some studies even suggest that few of these collagens can inhibit tumorigenesis, and that different levels of collagens have different effects [72–74]. This do suggest, that the turnover of fibroblast-collagens is important and relevant in the cancer setting and should be considered when exploring these collagens. Here we give an overview of these collagens and their contribution to tumorigenesis (Table 1).

**Table 1:** Overview of collagen type I, II, III, V, VI, XXIV and XXVII and their distribution in healthy tissue, cancer tissue, tumor promoting effects and liquid biomarker potential

Collagen type	Description	Reference
Collagen type I:		
• Tissue distribution	Main organic compound in bone. Also present in soft tissue.	[75]
• Tissue distribution in associated cancers	Major implications in bone cancer, and metastasis from bone to other solid tumors. Also described in breast, colorectal, ovarian, lung and pancreas cancer.	[21, 23, 24, 76–85]
• Tumor promoting effects	Associated with apoptosis, invasion, metastasis and proliferation.	[81–85]
• Liquid biomarker potential	Associated with bone metastasis in prostate and breast cancer patients. Increased in serum from colorectal, lung and pancreas cancer patients.	[21–25]
Collagen II		
• Tissue distribution	Main collagen in cartilage.	[86]
• Tissue distribution in associated cancers	Associated with chondrosarcoma	[88–90]
• Tumor promoting effects	Associated with cell death and survival	[88–90]
• Liquid biomarker potential	n/a	
Collagen type III		
• Tissue distribution	Primarily found in the vascular system, intestine, liver, skin and lung.	[86]
• Tissue distribution in associated cancers	Implications in breast, colorectal, HNSCC and pancreas cancer.	[21, 22, 34, 91–94]
• Tumor promoting effects	Associated with invasion, metastasis, migration and proliferation.	[73, 74, 82]
• Liquid biomarker potential	Augmented in serum from ovarian, breast, colorectal, melanoma and pancreas cancer patients. Able to predict pancreas cancer patients most likely to respond to treatment.	[21, 24, 94, 131]
Collagen type V		
• Tissue distribution	Primarily in same tissues as collagen type I and III.	[86, 95]
• Tissue distribution in associated cancers	Associated with breast cancer.	[96]
• Tumor promoting effects	Associated with tumor growth.	[96]
• Liquid biomarker potential	n/a	
Collagen type VI		
• Tissue distribution	Present in many tissues such as adipose, cartilage, skin, cornea, tendon, lung, skeletal muscle and dermis.	[97]
• Tissue distribution in associated cancers	Described in breast, colorectal, ovarian, gliomas, melanomas and pancreas cancer.	[98]
• Tumor promoting effects	Associated with apoptosis, drug resistance, inflammation, invasion, metastasis and proliferation.	[97–106]
• Liquid biomarker potential	Augmented in serum from melanoma and pancreas cancer patients.	[132, 133]
Collagen type XI		
• Tissue distribution	Distributed in low levels in skeletal muscle, trabecular bone, tendons, testis, trachea, articular cartilage, lung, placenta and brain.	[107–114]
• Tissue distribution in associated cancers	Extremely augmented in colorectal and HNSCC cancer. Also associated with breast, gastric, lung, ovarian and pancreas cancer.	[107–114]
• Tumor promoting effects	Highly implicated in CAF biology. Also associated with invasion, metastasis and proliferation.	[107–119]
• Liquid biomarker potential	n/a	
Collagen type XXIV		
• Tissue distribution	Distributed in ovaries, testis, liver, spleen, kidney, muscle and bone.	[120–122]
• Tissue distribution in associated cancers	Associated with HNSCC.	[123]
• Tumor promoting effects	Associated with cell differentiation.	[123]
• Liquid biomarker potential	n/a	
Collagen type XXVII		
• Tissue distribution	Expressed in the developing eyes, ears, lungs, heart and arteries.	[124–126]
• Tissue distribution in associated cancers	n/a	
• Tumor promoting effects	n/a	
• Liquid biomarker potential	n/a	

*HNSCC* Head and neck squamous cell carcinoma, *n/a* not available

### Type I collagen

Type I collagen is the most abundant collagen throughout the body. It is the major component of the bone and is present in blood vessels, cornea, sclera, tendon, ligaments and skin. It is the most common collagen in the IM, where it has key structural roles. Apart from its structural role, type I collagen possess important growth factor binding potential, and via its binding to a variety of proteins regulate cell homeostasis [75].

A number of studies have shown that type I collagen play a significant role in bone related diseases, inclusive bone cancer and cancer-related bone metastasizes. Especially the turnover of type I collagen has shown to be important [76–79].

Type I collagen is also dysregulated in other solid tumor types (than bone cancer) and can affect tumor cell behavior. Compared to healthy tissue, the amount of type I collagen is augmented in pancreas, colorectal, ovarian, breast and lung cancer [21, 23, 24, 80].

Pancreas cancer cells exposed to type I collagen show increased proliferation, are less responsive to apoptosis, secrete higher amounts of TGF-β and show a strong reduction in E-cadherin expression [81–83]. Interestingly, Gao et al. found that tumor cells, in mouse breast tumor tissue, show high proliferative activity when located adjacent to type I collagen, whereas cells not in contact with type I collagen are quiescent [84].

Type I collagen has also been shown to affect metastasis, as exposure to type I collagen results in more invasive behavior in tumor cells [82]. In an in vivo breast cancer model, with accumulated type I collagen distribution, the amount of circulating tumor cells was increased compared to the amount in wild type mice. Moreover, the metastatic lesions were larger than in wild type [85].

### Type II collagen

Type II collagen is the main collagen in cartilage, where it constitutes 80% of the total collagen content [86]. Within the joint, it provides stability and resiliency to stress [86]. Forty percent of all bone cancers originates from cartilage, however bone cancers accounts for less than 0.2% of all cancers [87] and therefore very little is known about type II collagen and its relation to cancer. However, a few studies have shown that type II collagen can affect cell behavior and that the type II collagen fragment PIIBNP can inhibit osteoclast survival and induce cell death in tumor cells [88–90].

### Type III collagen

Type III collagen is the second most abundant collagen and is often distributed close to type I collagen. It is primarily found in vascular systems, intestine, liver, skin and lung [86]. Like type I collagen, type III collagen distribution is augmented in many cancer diseases such as head and neck squamous cell cancer (HNSCC), breast, pancreas and colorectal cancer [21, 22, 34, 91–94]. In colon cancer, the distribution of type III collagen is especially augmented next to neovascular tissue [34, 91].

Pancreas cancer cells grown on type III collagen show increased proliferation, migration and decreased expression of E-cadherin [82]. Moreover, type III collagen is involved in invasion and metastasis of glioblastoma cells. These cells show high invasion and migration response when exposed to type III collagen and antibodies against type III collagen inhibit these processes [73]. Another study, report that collagen III is one of few genes that are modified, when invasive prostate cancer cells interact with bone marrow stromal cells, within the bone microenvironment. This interaction is crucial for the metastasis process, which further suggests an involvement of type III collagen in invasion and metastasis [74].

### Type V collagen

Type V collagen is a minor fibrillary collagen expressed in same tissues as collagen I and III, and helps in the formation of tissue specific matrices [86, 95]. Especially, the a3 chain of type V collagen has shown to be involved in cancer biology. When injecting breast tumor cells into mice deficient of the a3 chain in collagen 5 (Col5a3^−/−^) tumor growth is reduced and survival prolonged compared to wildtype littermates [96]. In addition, Col5a3^−/−^ cancer cells injected into Col5a3^−/−^ and Col5a3^+/+^ mice prolonged survival significantly in both genotypes compared to injection of cells containing the collagen V a3 chain [96]. Thus, these two examples suggest that the presence of the collagen V a3 chain promote tumor growth.

### Type VI collagen

Type VI collagen is present in many tissues such as adipose, cartilage, skin, cornea, tendon, lung, skeletal muscle and dermis. It is located near the BM where it functions as a mediator between the BM and IM via its many binding sites in both matrices. It can bind to a wide variety of proteins such as type I, II, IV, XIV collagen, integrin’s, fibronectin, tenascin etc. Type VI collagen has many roles covering structural purposes to more cell-specific functions including regulation of apoptosis, proliferation, differentiation and maintenance of cell stemness [97]. Collagen VI expression is increased in many human tumors such as glioblastomas, melanomas, ovarian, pancreatic, breast and colon cancer [98]. In vitro and in vivo studies have shown that collagen VI increase proliferation and decrease apoptosis in breast, melanoma and glioblastoma cell lines [97, 98]. Apart from its direct stimulatory effects on tumor cells, collagen VI also affects the tumor microenvironment by promoting angiogenesis and inflammation [98, 99]. Collagen VI deficiency (col6^−/−^) inhibit endothelial cell growth and sprouting of new vessels in a melanoma mouse model. Regarding inflammation, macrophages has been shown to produce type VI collagen, which in this context, modulate cell-to-matrix and cell-to-cell interactions [100]. Lastly, type VI collagen has shown to affect the invasion-profile of glioblastoma and lung-cancer cell [101, 102].

A number of studies have shown, that the a3 chain and the C5 domain of the a3 chain, also called endotrophin is involved in many hallmarks in cancer such as promoting proliferation, angiogenesis, metastasis and chemotherapy resistance. Type VI collagen a3 is distributed in high amounts in lung, ovarian, pancreatic, colon and breast cancer tissues [98]. Endotrophin has been found to promote metastasis in breast cancer and recruit endothelial cells to the tumor microenvironment [99]. This study also reported that endotrophin facilitate tumor cell proliferation and metastasis through TGF-β activation as well as promote inflammation in the tumor microenvironment by upregulating inflammatory markers such as interleukin-6 and TNF-a [99]. In the context of chemotherapy resistance collagen VI a3 is one of the most highly expressed genes in cisplatin and oxaliplatin resistant ovarian cancer cells [103, 104]. In addition, endotrophin is highly upregulated in cisplatin resistant breast tumor cells, and inhibition of endotrophin lead to cisplatin sensitivity in a breast tumor mouse model [105]. Metallothioneins, which are associated with cisplatin resistance, are highly upregulated in breast cancer cells treated with collagen VI, which could be one of the explanations for the chemotherapy resistance, as suggested by Iangyar et al. [106].

### Type XI collagen

Type XI collagen is present in low levels in skeletal muscle, trabecular bone, tendons, testis, trachea, articular cartilage, lung, placenta and in the brain. It is a minor fibrillar collagen, which co-polymerize with type II collagen and type IX collagen. In cartilage, it is extremely important for proper function, as absence of type XI collagen lead to abnormal thickening of the tissue. Collagen XI has long been suspected to be of high impact in cancer formation, and especially the a1 chain of collagen XI has shown to be an important player in various cancer diseases. The gene signature of type XI collagen is upregulated in breast, gastric, pancreatic, and non-small lung cancer. Interestingly, in both colon and HNSCC the expression is extremely increased with almost no expression in healthy controls [107–114]. Knock down of type XIa1 collagen in HNSCC and ovarian cancer cell lines, significantly decrease proliferation, invasion and migration compared to controls, which highlight type XI collagens importance in cancer [107, 115]. In breast and ovarian cancer collagen XIa1 has also been associated with resistant to chemotherapy [116, 117].

Type XI collagen is highly associated with CAFs. CAFs originating from HNSCC, lung cancer and pancreas cancer tissue express higher levels of collagen XIa1 than cells arrived from healthy tissue [107, 110, 118]. In ovarian and pancreatic cancer CAFs strongly stain for collagen XIa1, compared to no staining in epithelial cancer cells and healthy tissue [110, 119].

### Type XXIV

Type XXIV collagen is expressed in ovaries, testis, liver, spleen, lung, kidney, muscle and bone and is located close to type I and V collagen [120–122].

As with type II collagen very little is known regarding type XXIV collagen in relation to cancer. Type XXIV collagen has been associated with osteoblast differentiation with the expression increased in tumor tissue from patients suffering from HNSCC [123].

### Type XXVII

Like type XXIV collagen, type XXVII is a relatively poorly characterized collagen. During embryogenesis in mice COL27A is expressed in the developing eyes, ears, lungs, heart and arteries [124, 125]. However, in adults it is primarily expressed in cartilage, and is therefore thought to play a role in the development phases [126]. Type XXVII collagens role in cancer is yet to be investigated.

### Stromal derived biomarkers in clinical cancer research

A number of studies have investigated the possibility of using CAFs as prognostic markers in different cancer diseases. The most widely CAF biomarkers used for this are a-SMA, Vimentin, collagen XIa, fibronectin, FSP-1 and FAP. In esophageal cancer a-SMA and FSP-1 positive staining correlates with larger tumor size, advanced T-stage and shorter survival [127]. FAP is highly expressed in CAFs and present in many different cancer types, and has been associated with shorter survival in lung, esophageal and breast cancer [47, 128]. CAFs are very complex cells and the CAF markers used today display cellular overlaps, and have to be used in combinations [129]. Therefore, developing specific CAF biomarkers or biomarkers measuring CAF activity, i.e. disease progression, should be of high priority.

The existing CAF biomarkers are mainly based on immunohistochemistry, which rely on tissue biopsies. Although such tissue biomarkers are still the golden standard for tumor characterization, there are several benefits of developing biomarkers based on liquid biopsies (e.g. serum, plasma, urine). Besides being non-invasive, cost-effective and highly repeatable, liquid biopsies are also a real-time representative for the entire tumor heterogeneity, and not just a snapshot of the tumor tissue here and now [130].

The formation and degradation of fibroblast-derived collagens, during desmoplasia, are mediated by CAFs [33]. Thus, collagen fragments could be a measure of CAF activity. Interestingly, formation- and degradation products, in serum, from fibroblast-derived collagens show diagnostic and prognostic value. Degradation products from collagen I are significantly increased in colorectal cancer and able to differentiate stage IV colorectal cancer from stage I-III. [24]. The same trend is seen in ovarian, breast, lung and pancreas cancer patients, where degradation products from collagen I can distinguish cancer patients from healthy controls [21–23]. Moreover, a strong association between formation products from collagen I and the amount of bone metastasizes is seen in prostate and breast cancer [25]. Collagen III formation and degradation products are elevated in ovarian and breast cancer patients, and capable of distinguishing cancer patients from healthy controls [21]. This is also shown for colorectal cancer where collagen III products are significantly elevated and correlate with tumor stage [24]. Interestingly, the ratio of formation and degradation markers of collagen III has shown to be capable of predicting pancreas patients most likely to respond to the hyaluronan targeting drug PEGPH20 (pegvorhyaluronidase alfa) [131]. In addition, a high ratio predicts increased overall survival in melanoma patients [94]. Lastly, serum levels of collagen VI are increased in melanoma and pancreatic cancer patients [132, 133].

Another potential role of collagen biomarkers are related to anti-TGF-β therapies that are emerging as novel treatments options, in particular in the immuno-oncology setting. TGF-β is a complex molecule with many roles in cancer [103, 134] amongst others TGF-β stimulate CAFs to produce collagens [28, 135]. Hence collagen turnover fragments may be predictive of a TGF-β driven phenotype and hence be used to identify patients benefiting from such treatment. In addition, these collagen biomarkers may be used to monitor on target effects of TGF-β and reveal valued information on mode of action of the compound investigated. A recent study has shown that the assembly of collagens can trap T-cells preventing them to access the tumor, and induce T-cell dependent cell death [136]. This complicate the use of immune therapy and could be a reason to why only a subset of patients respond to therapy. In the last mentioned study, the occurrence of TGF-β producing fibroblasts was strongly associated with lack of therapy response [136]. In this respect, collagen levels have the potential to be used as precision medicine to select patients most likely to respond to treatment.

## Conclusion

Alterations in tissue microarchitecture is a hallmark of cancer driven by CAFs and the associated deposition of collagens in the tumor stroma, which amongst other things leads to desmoplasia, poor prognosis and therapy resistance. In this review we have highlighted the link between CAFs, the fibrillar collagens produced by CAFs, and tumorigenesis. We provide a rationale for studying CAF-derived collagens in greater detail, to improve the understanding of tumor biology and patient characteristics. Lastly, we argue that a major biomarker potential lies in the fact that these collagen products can be measured in a liquid biopsy, providing a surrogate measure of desmoplasia and CAF activity. Future biomarker research should focus on implementing such biomarker tools in the clinical setting for phenotyping of cancer patients and potentially for predicting and monitoring response to treatment.

## Abbreviations

BM: Basement membrane
CAF: Cancer associated fibroblast
ECM: Extracellular matrix
FAP: Fibroblast activating protein
FSP-1: Fibroblast specific protein 1
HNSCC: Head and neck squamous cell cancer
IM: Interstitial matrix
LOX: Lysyl oxidase
MMP: Metalloproteinases
n/a: Not available
TGF-β: Transforming growth factor beta
α-SMA: alpha-smooth muscle actin
